# Reply to Carrera-Gil et al. Comment on “Sum of Skinfold-Corrected Girths Correlates with Resting Energy Expenditure: Development of the NRG_CO_ Equation. *Nutrients* 2024, *16*, 3121”

**DOI:** 10.3390/nu17111811

**Published:** 2025-05-27

**Authors:** Jorge L. Petro, Diego A. Restrepo-Botero, Camilo A. Rincón-Yepes, Katherine Franco-Hoyos, Alejandra Agudelo-Martínez, Luis A. Cardozo, Leidy T. Duque-Zuluaga, Jorge M. Vélez-Gutiérrez, Roberto Cannataro, Andrés Rojas-Jaramillo, Richard B. Kreider, Diego A. Bonilla

**Affiliations:** 1 Research Division, Dynamical Business & Science Society—DBSS International SAS, Bogotá 110311, Colombia; jlpetros@dbss.pro (J.L.P.); restrepo.diego@uces.edu.co (D.A.R.-B.); rincon.camilo@uces.edu.co (C.A.R.-Y.); lduque@dbss.pro (L.T.D.-Z.); jorge.velez@arthros.com.co (J.M.V.-G.); r.cannataro@gmail.com (R.C.); andres.rojasj@udea.edu.co (A.R.-J.); 2 Research Group in Physical Activity, Sports and Health Sciences (GICAFS), Universidad de Córdoba, Monteria 230002, Colombia; 3 Grupo de Investigación NUTRAL, Facultad Ciencias de la Nutrición y los Alimentos, Universidad CES, Medellín 050021, Colombia; kfranco@ces.edu.co (K.F.-H.); magudelo@ces.edu.co (A.A.-M.); 4 Research and Measurement Group in Sports Training (IMED), Faculty of Health Sciences and Sports, Fundación Universitaria del Área Andina, Bogotá 111221, Colombia; lcardozo11@areandina.edu.co; 5 ARTHROS Centro de Fisioterapia y Ejercicio, Medellín 050012, Colombia; 6 Department of Pharmacy, Health and Nutritional Sciences, University of Calabria, 87036 Rende, Italy; 7 Research Group of Sciences Applied to Physical Activity and Sport, Universidad de Antioquia, Medellín 050010, Colombia; 8 Exercise & Sport Nutrition Laboratory, Human Clinical Research Facility, Texas A&M University, College Station, TX 77843, USA; rbkreider@tamu.edu; 9 Hologenomiks Research Group, Department of Genetics, Physical Anthropology and Animal Physiology, University of the Basque Country (UPV/EHU), 48940 Leioa, Spain

We appreciate the comments and interest in our study [1] by Carrera-Gil et al. (2025) [2], particularly regarding (i) the proportion of estimates differing by more than 10% from the measured value; (ii) participants’ adherence to standardized procedures during indirect calorimetry, along with the absence of nitrogen correction and other outcomes; (iii) potential technical errors and practicality limitations of the anthropometric variables used as regressors in the new NRG_CO_ equation (girths and skinfolds); and (iv) the overall limitations and inaccuracy of predictive equations.

Regarding the suggestion to report the estimates that differ by more than 10% from the measured resting energy expenditure (REE), we acknowledge that the ratio of measured to estimated REE has been used as a proxy indicator of energy availability, which is of interest for various clinical applications at the individual level (e.g., nutrition across different contexts to avoid energy deficiency) [3]. In our analysis, we chose to present robust metrics within the context of predictive equations at the group level, such as the mean bias (91.5 kcal; 95% limits of agreement: −690, 873) and the standard error of the estimate (SEE = 280.52 kcal), along with Bland–Altman plots, which allow visualization of the error dispersion and bias trend across a wide range of REE values [1]. Also, we reported a coefficient of determination (R^2^) of 0.57 for the developed model, indicating that 57.0% of the variance in measured REE values is explained by the model. This metric provides a clear measure of the model’s utility at the group level, reflecting its ability to describe general trends in the studied population. In response to the observation, we calculated the proportion of individuals whose estimated REE differed by more than 10% from the measured value, which was 0.540 (54.0%). This highlights that, while the model explains a substantial portion of the variance at the group level, there is variability at the individual level that warrants consideration. We agree with the researchers that the individual-level approach (e.g., identifying subjects with >10% error) could be particularly useful for personalized calorie prescription and for assessing whether the equation is suitable for individual use.

However, we would like to emphasize that the variance explained by the model (R^2^) and the proportion of individuals with >10% error are complementary metrics that, in homogenous samples, are inversely correlated, as the researchers are aware. In our case, the relatively high proportion of individuals with >10% error (54.0%) aligns with the variance explained by the model (R^2^ = 0.570), suggesting that the model captures a significant portion of the variability but also leaves room for improvement in individual-level accuracy. Readers should be aware that a model could have a moderate or high R^2^ but still have a high proportion of errors >10% if the errors are concentrated in a few individuals with very large errors (this is frequent in heterogeneous samples). Furthermore, we fully agree with O’Neill et al. (2023) [3] that longitudinal monitoring is a more suitable approach for detecting REE suppression and evaluating energy availability at the individual level. As stated in their meta-analysis, “a more suitable use may be in longitudinal monitoring, and interpretation of directly measured REE and body composition. REE values relative to body mass and/or fat-free mass can then be compared to detect the suppression of REE.”. As we highlight in our updated version of the 4Rs of sports nutrition [4] (unpublished article in press in *Life* by MDPI), readers and practitioners should note that the strict definition of low energy availability is often impractical in real-world settings. This is because it requires highly accurate measurements of dietary energy intake, energy expenditure (which varies daily based on training load), and body composition. In practice, accurate measurements of energy intake and fat-free mass are challenging. Therefore, the need for intervention is typically determined based on the identification of symptoms and the assessment of biomarkers, such as hematological parameters and selected plasma hormones known to fluctuate with low energy availability. Our team strongly supports this perspective and considers longitudinal individual monitoring to be of greater value for practical applications, particularly in athletic populations where detecting REE suppression is critical [3].

Regarding more detailed information on fasting conditions and physical activity before measurement, in our study, these factors were controlled according to established methodological recommendations by the American Dietetic Association [5]. Although we cite it accordingly, we share this for clarity and reproducibility in clinical practice (Table 1). Researchers provided these instructions to participants in our study via WhatsApp 4–7 days in advance (when scheduling the evaluation) and confirmed compliance immediately before the indirect calorimetry assessment. The initial rest period and environmental conditions were carefully controlled in the laboratory setting. It is worth mentioning that more recent references align with these recommendations [6]. Similarly, measures of VCO_2_, VO_2_, and the respiratory quotient (RQ) were recorded following this standardized protocol but were not reported in the original article, as they do not correspond to the primary outcome of the study [1]. These data will be reported in future publications as part of the NRG project.

We recognize that the absence of nitrogen correction in protein oxidation calculations represents a limitation of the study, as its measurement allows for a more accurate estimation of energy balance and the contribution of protein oxidation to resting energy expenditure. However, as stated in our previous protocol (NCT05832710), this evaluation was not included in the project due to laboratory limitations. We hope to incorporate this analysis in future phases (budget permitting) to complement metabolic assessment and strengthen data interpretation in specific populations.

Regarding the coefficient of determination of our equation (R^2^ = 0.57 [0.37, 0.65]), we acknowledge that it is lower than that of other equations, such as the Mifflin–St. Jeor equation with three predictors (R^2^ = 0.71). For clarity, readers should note that Mifflin et al. (1990) [7] tested several models in their seminal study, some of which had R^2^ values ≤ 0.56 and are frequently used in online energy expenditure calculators due to their simplicity (one or two predictors). As discussed in our article, the new NRG_CO_ equation represents an initial effort to develop a model specifically tailored to the Colombian population, where no prior equations had been created. Therefore, we emphasize that while the coefficient of determination is relevant, it should not be the sole criterion for evaluating the usefulness of a predictive equation, as its applicability and agreement with experimentally measured values are equally important. In this regard, our article highlights that several validated equations in other populations have shown determination coefficients below 0.5 and continue to be used for estimating REE [8]. Consequently, we consider this new equation to be a viable alternative in this context, and its implementation in future studies will allow for continued refinement of its accuracy and applicability in the Colombian population. In addition, we appreciate the researchers for emphasizing the applicability of the proportion of individuals with >10% error and encourage its use as a practical recommendation for personalized interventions and the detection of REE suppression (while more robust methods are developed).

Regarding the claim that the measurement of skinfold thickness and corrected girths compromises the reproducibility and accuracy of our equation, we do not fully agree with this statement. The correct application of any technique, including kinanthropometry, requires certified evaluators with experience and rigorous control of key aspects, such as determining intra- and inter-evaluator error. In this regard, our measurements were conducted following validated protocols and carried out by trained personnel, ensuring their reliability. It is worth mentioning that our initial hypothesis considered the estimation of REE based on simple anthropometric variables associated with musculoskeletal mass (corrected girths and sum of skinfolds, the latter as an indicator of whole-body adiposity). Indeed, as a novel contribution, this study is the first to introduce and evaluate the use of the sum of three corrected girths (∑3CG: arm, thigh, calf) and the sum of three breadths (∑3D: humerus, bi-styloid, femur) in developing a model to estimate REE. Notably, we found that both ∑3CG and ∑3D demonstrated significant positive correlations with REE, highlighting their potential utility in predictive models.

Furthermore, incorporating the absolute values of the sum of skinfolds in the simplest model provides a viable alternative in contexts where access to advanced technologies, such as DXA, is limited due to its high cost and the impracticality of conducting repeated measurements in applied studies. This is particularly the case for Colombian facilities for exercise and nutrition assessment, where DXA is constrained to research laboratories and private clinics that reduce its accessibility. Therefore, we encourage professionals in health and exercise science to enhance their training in these techniques through certifications and specialized training programs (i.e., ISAK certifications), ensuring precise and reproducible measurements of absolute values in both research and clinical practice. In this sense, our equation is a valuable and applicable option in our country, especially when compared to equations developed in other populations (i.e., with different characteristics or conditions) or those requiring technologies that are difficult to access in clinical and sports settings.

We acknowledge that estimating REE (or any other energy-related variable) remains challenging and that developing predictive equations is an ongoing process. Our study represents an initial step in developing and validating models specific to the Colombian population. Ongoing research within the NRG Project will continue refining these equations, enhancing their applicability and accuracy with larger samples and in diverse populations (e.g., children and adolescents).

Finally, our results have been reported transparently, strictly adhering to the established guidelines from study design, registration, and methodological protocols to approval by the ethics committee. We appreciate the time and effort dedicated to reviewing our work and hope this exchange enriches the scientific discussion on the topic.

## Figures and Tables

**Table 1:** Evidence-based guidelines for measurement of REE with indirect calorimetry.

Criteria	Healthy Adults
Fasting	Minimum fast 5 h after meals or snacks (Grade II) ^a^, 4 h after small meal if longer fast is clinically inappropriate (Grade II).
Alcohol ingestion	Minimum abstention from alcohol for 2 h (Grade III).
Nicotine ingestion	Minimum abstention from nicotine for 2 h (Grade III).
Caffeine ingestion	Minimum abstention from caffeine for 4 h (Grade II).
Rest periods	Rest 10–20 min (Grade III).
Physical activity restriction	Minimum abstention from moderate cardiovascular or strength exercise for 2 h before test (Grade II), for vigorous resistance exercise abstention of at least 14 h (Grade III). All participants in our study were instructed to avoid strenuous physical exercise for at least 24 h.
Environmental conditions	Allow a room temperature of 20–25 °C (Grade III) in comfortable conditions (Grade V).
Gas collection devices and steady-state conditions	Use rigorous adherence to prevent air leaks (Grade III). Discard the initial 5 min. Then, achieve a 5 min period with ≤10% CV ^b^ for VO_2_ ^c^ and VCO_2_ ^d^ (Grade II).
No. of measures/24 h	Achieve a steady state and one measure is adequate; if not, two to three nonconsecutive measures improve accuracy (Grade II).
Repeated measures (daily to monthly variation)	Repeated measures vary 3–5% over 24 h (Grade II) and vary up to 10% over weeks to months (Grade II).
Respiratory quotient (RQ)	RQ measures < 0.70 or >1 suggest protocol violations or inaccurate gas measurements (Grade II).

^a^ Grade I: strong, consistent evidence; Grade II: somewhat weaker evidence and disagreement among authors may exist; Grade III: limited design quality; Grade IV: professional opinion only, no clinical trials; Grade V: no available studies. ^b^ CV = coefficient of variation (standard deviation × [mean of individual replicate measures] × 100). ^c^ VO_2_: oxygen consumption. ^d^ VCO_2_: carbon dioxide production. Reproduced from: Compher et al. [5].
